# The Evolving Reduction of Vancomycin and Daptomycin Susceptibility in MRSA—Salvaging the Gold Standards with Combination Therapy

**DOI:** 10.3390/antibiotics9110762

**Published:** 2020-10-30

**Authors:** Taylor Morrisette, Sara Alosaimy, Jacinda C. Abdul-Mutakabbir, Razieh Kebriaei, Michael J. Rybak

**Affiliations:** 1 Anti-Infective Research Laboratory, Department of Pharmacy Practice, Eugene Applebaum College of Pharmacy and Health Sciences, Wayne State University, Detroit, MI 48201, USA; Gx6737@wayne.edu (T.M.); salosaimy@wayne.edu (S.A.); gr8442@wayne.edu (J.C.A.-M.); R.Kebriae@wayne.edu (R.K.); 2 Division of Infectious Diseases, Department of Medicine, Wayne State University, Detroit, MI 48201, USA; 3 Department of Pharmacy, Detroit Receiving Hospital, Detroit, MI 48201, USA

**Keywords:** vancomycin, daptomycin, MRSA, combination therapy

## Abstract

Methicillin-resistant *Staphylococcus aureus* (MRSA) is associated with substantial morbidity and mortality. Vancomycin (VAN) has been used as the gold standard treatment for invasive MRSA infections for decades but, unfortunately, the reliance of VAN as the primary treatment option against these infections has led to a reduction in VAN susceptibility in MRSA isolates. Although daptomycin (DAP) is another common treatment option against invasive MRSA infections, it has been shown that the development of VAN resistance can lead to DAP nonsusceptibility. VAN or DAP backbone regimens in combination with other antibiotics has been advocated as an alternative approach to improve patient outcomes in VAN/DAP-susceptible infections, enhance outcomes in infections caused by isolates with reduced VAN/DAP susceptibility, and/or prevent the emergence of VAN/DAP resistance or further resistance. A peer-reviewed literature search was conducted using Medline, Google Scholar and PubMed databases. The primary purpose of this review is to describe the mechanisms and epidemiology of MRSA isolates with a reduction in VAN and/or DAP susceptibility, evaluate in vitro and in vivo literature describing combination therapy (CT) against MRSA isolates with reduced VAN and/or DAP susceptibility and describe studies involving the clinical outcomes of patients treated with CT against invasive MRSA infections.

## 1. Introduction

Methicillin-resistant *Staphylococcus aureus* (MRSA) is a predominant pathogen that causes human infections in both community and hospital settings [1]. Although vancomycin (VAN) is the most commonly used antibiotic to treat invasive MRSA infections, increased utilization of this antimicrobial has led to the emergence of clinical MRSA isolates with reduced susceptibility to VAN [1]. Additionally, the clinical outcomes of heterogeneous VAN-intermediate *S. aureus* (hVISA) and VAN-intermediate *S. aureus* (VISA) infections are poor and treatment options for *S. aureus* infections with reduced susceptibility to VAN are limited [2,3].

In addition to VAN, there are some viable alternative antimicrobial agents with in vitro activity against MRSA, such as daptomycin (DAP), teicoplanin, linezolid, ceftaroline (CPT), trimethoprim/sulfamethoxazole (TMP/SMX), tigecycline and quinupristin/dalfopristin [4]. Importantly, these alternative agents have not proven to be superior to VAN in invasive MRSA infections. Although DAP is becoming an addition to the “gold standard arsenal” of invasive MRSA treatment, DAP resistance has been shown to positively correlate with VAN resistance, with a recent report concluding that a mutation in *mprF* was responsible for this cross-resistance [5,6,7]. Combination therapy (CT), commonly with beta-lactams (BL) or other anti-MRSA agents, may provide an alternative option to reduce the emergence of resistance and for combating *S. aureus* infections caused by phenotypes with reduced susceptibility to VAN and/or DAP to improve patient outcomes [8,9].

This review begins with an overview of the mechanistic basis and epidemiology of hVISA, VISA, VAN-resistant *S. aureus* (VRSA) and DAP-non-susceptible (DNS) strains. We then focus on in vitro and in vivo studies evaluating the impact of CT on these resistant phenotypes and conclude with an evaluation of health outcomes studies analyzing CT against MRSA (although not all MRSA outcomes studies were evaluated against organisms with increased resistance to VAN and/or DAP and clinically relevant studies evaluating methicillin-susceptible *S. aureus* (MSSA) will also be described).

## 2. Methods

A search of peer-reviewed literature was conducted using Medline, Google Scholar and PubMed databases between 1935 and 2020. Search terms included *S. aureus*, MRSA, hVISA, VISA, VRSA, DNS, combination therapy, VAN combination therapy, DAP combination therapy and beta-lactams. The reference lists associated with the retrieved articles were also examined for pertinent articles. If the authors were unable to translate the text to English within the retrieved articles, they were excluded.

## 3. Beta-Lactam Resistance

### Methicillin-Resistant Staphylococcus aureus

*Staphylococcus aureus* is a Gram-positive and coagulate-positive pathogen that has the capability of acquiring resistance to nearly all antibiotics [1]. Following the discovery and utilization of penicillin, *S. aureus* isolates that became resistant were identified within two years [10,11]. Methicillin, a semisynthetic penicillin, was developed to overcome the beta-lactamase-mediated resistance against penicillin; however, methicillin-resistant strains began to emerge in 1961, following its clinical use [12]. According to the Clinical and Laboratory Standards Institute (CLSI), MRSA isolates currently demonstrate minimum inhibitory concentrations (MIC) values of ≥ 4 mg/L against oxacillin, while the European Committee on Antimicrobial Susceptibility Testing (EUCAST) do not list a specific breakpoint and state that “*S. aureus* with oxacillin MIC values >2 mg/L are mostly methicillin resistant” [4,13]. The mechanism of methicillin (and most other BL) resistance is via *mecA*, an acquired gene that encodes penicillin-binding protein 2a (PBP2a), which has a low affinity for nearly all BL [14]. Although once confined to mainly healthcare settings, community spread of MRSA has emerged and, compared to methicillin-susceptible strains, MRSA infections typically lead to higher mortality rates, longer hospital length of stays and increased healthcare costs [1,15,16]. Importantly, in hospital settings, strains with phenotypic resistance to methicillin typically occur in 25–50% of isolated strains [17]. Although the epidemiology of MRSA has constantly changed over the years, the Centers for Disease Control and Prevention currently classify MRSA as a serious threat, while the World Health Organization currently lists MRSA on their priority 2: high list and novel therapeutic options to manage this pathogen are still needed [18,19].

## 4. Vancomycin and Daptomycin Resistance

### 4.1. Heterogeneous Vancomycin-Intermediate Staphylococcus aureus and Vancomycin-Intermediate Staphylococcus aureus

According to the CLSI, VISA isolates demonstrate MIC values of 4–8 mg/L, whereas hVISA strains appear to be susceptible to vancomycin with MIC values within the susceptible range via traditional susceptibility testing methods but containing subpopulations of VISA cells, while EUCAST does not list MIC values for isolates to be considered hVISA/VISA [4,13]. The most precise method for the determination of heteroresistance is referred to as the population analysis profile/area under the curve (PAP/AUC) ratio [20,21,22]. This method utilizes agar plates with varying concentrations of VAN and defines the area under the log_10_ CFU/mL counts versus concentration curve (AUC) [21,22]. In clinical settings, hVISA cases typically go undetected owing to suboptimal (or lack of) screening programs and the inherent labor-intensive nature of current hVISA testing protocols. The first strains with reduced susceptibility to VAN were isolated in 1996 [23,24,25]. Strains with the hVISA/VISA phenotype have demonstrated lower growth rates, thicker cell walls, increased residues of D-alanine-D-alanine (VAN primary binding site) and a clogging mechanism that affects VAN’s mechanism when compared to their susceptible counterparts as VAN resistance is accompanied by mutations affecting cell wall biosynthesis [23,26]. The hVISA stage is reported to precede the development of VISA, especially with further exposure to VAN. Therefore, at MIC values >1 mg/L, alternative approaches such as other monotherapies or CT should be considered [6,23,27]. Importantly, the pooled prevalence of hVISA was reported to be 6.05% and VISA to be 3.01% in 99,042 and 68,792 MRSA strains, respectively, in 2015 and the prevalence of both phenotypes has been steadily increasing [27,28,29]. Furthermore, hVISA and VISA infections are associated with VAN treatment failures, persistent infections and sub-optimal patient outcomes [2,3].

### 4.2. Vancomycin-Resistant Staphylococcus aureus and Daptomycin-Nonsusceptible Staphylococcus aureus

According to CLSI and EUCAST, VRSA isolates demonstrate MIC values of ≥ 16 mg/L and > 2 mg/L, respectively [4,13]. Vancomycin resistant *Staphylococcus aureus* infections are uncommon in current practice and, thus, there is no standardized treatment for *Staphylococcus aureus* infections with the VRSA phenotype [30]. The first VRSA strain isolated from a patient at the Detroit Medical Center was reported in 2002, with a mechanism of resistance that is different from that of hVISA/VISA [31]. The mechanism of VRSA resistance involves the *vanA* operon that was originally transferred from VAN-resistant enterococci plasmids. The *vanA*-mediated resistance in VAN leads to hydrolysis of D-alanine-D-alanine and synthesis of new peptidoglycan precursors that VAN does not bind to (D-alanine-D-lactate) [32,33]. Thus far, there has been 14 VRSA isolates from humans reported in the United States [34].

The potent staphylocidal activity of DAP has become a mainstay of anti-MRSA therapy, commonly utilized in instances where resistance has developed to VAN or patients were not responding to therapy; as shown in *S. aureus* strains with either the hVISA, VISA or VRSA phenotypes [35]. Consequently, both in vitro and in vivo research have shown parallel increases in DAP MICs, when compared to that of VAN MICs in staphylococcal isolates, presenting as the aforementioned DNS strains [5]. According to CLSI and EUCAST, DNS strains exhibit MICs > 1 mg/L [4,13]. Reduced susceptibility to DAP has been attributed to a number of genetic mutations most notably in the *mprF* locus, which correlate with an alteration in bacterial cell walls, surface charge and the cell membrane. Noting this mechanistic basis, innovative approaches to manage reduced DAP susceptibility, including CT with additional antistaphylococcal therapy, have been applied to enhance and/or retain DAP activity [36,37]. Important to note, a potential undesirable consequence of the hVISA and/or VISA phenotypes is that the abnormalities in the cell envelope of these strains can lead to higher DAP MICs, perhaps due to repulsion of the cationic daptomycin-calcium complex as a result of altered membrane surface charge [38,39].

## 5. In Vitro and In Vivo Studies

### 5.1. Heterogeneous Vancomycin-Intermediate Staphylococcus aureus and Vancomycin-Intermediate Staphylococcus aureus

There have been numerous in vitro studies conducted analyzing the potential for synergy with VAN in combination with various BL agents against resistant phenotypes of *S. aureus*. Werth and colleagues performed time-kill assays (TKA) utilizing VAN plus oxacillin that demonstrated synergy in 1/5 hVISA and 3/5 VISA strains, while combination of VAN plus CPT was synergistic against 4/5 hVISA and 5/5 VISA strains. Furthermore, VAN + CPT demonstrated bactericidal activity in 2/5 hVISA and 3/5 VISA strains [38]. Another comparative study with 25 hVISA and 25 VISA isolates tested MIC values against VAN alone and in combination with CPT. The values showed up to 16-fold and 64-fold reduction in MIC values for VAN-CPT combination in hVISA and VISA strains, respectively, in comparison to VAN alone. The authors also tested this combination in an in vitro one-compartment pharmacokinetic/pharmacodynamic (PK/PD) model for seven days. As expected, VAN monotherapy was not effective against any of the hVISA or VISA isolates, while the combination of VAN-CPT reached detection limit within ~48–72 h [39]. Hagihara and colleagues performed an in vitro one-compartment PK/PD model with humanized antimicrobial exposures with the combination of VAN-cefazolin against hVISA and VISA isolates. Based on this report, CT demonstrated improved antibacterial activity against both strains when compared to VAN monotherapy; although, this difference was primarily driven by more rapid reductions in bacterial inoculum [40]. Additionally, combination of VAN with cefazolin, cefepime, CPT and nafcillin in combination MIC testing demonstrated a 2–64-fold reduction compared to VAN alone and 24 h time kill experiments displayed enhanced antibacterial activity with CT against hVISA and VISA isolates [41]. Another report evaluated VAN combinations with various cephalosporins (cefazolin, cefmetazole, cefotaxime and cefepime) against eight hVISA and seven VISA clinical isolates. Following combination MIC testing, MIC mean fold reductions for hVISA and VISA strains were 1.81–3.83 and 2.71–9.33, respectively. Furthermore, the addition of cephalosporins to VAN reduced the AUC/Mu3 AUC ratios for hVISA and VISA strains between 1.81–2.02 and 2.37–2.85-fold, respectively [20]. Zheng and colleagues concluded that VAN plus BL (cefazolin, piperacillin-tazobactam) limited hVISA strain progression to VISA throughout a 28-day exposure and was associated with decreased cell wall thickness [42]. Climo and colleagues performed an analysis of the combination of VAN with nafcillin against strains of glycopeptide intermediate *S. aureus* (GISA) in an experimental endocarditis model in rabbits and found reductions of ~3–4 log-_10_ CFU/g in aortic valvular vegetations compared to VAN monotherapy [43]. Combinations of VAN plus oxacillin in time-kill experiments against 23 hVISA strains exhibited synergistic antibacterial effects in comparison to each agent alone [44]. Furthermore, piperacillin-tazobactam and oxacillin have been tested in combination with VAN and have demonstrated enhanced antibacterial activity in time-kill experiments against VISA strains [45]. Another evaluation performed by Werth and colleagues performed a one-compartment PK/PD model comparing VAN + CPT to either agent as monotherapy against isolate D592 (hVISA and DAP-susceptible) and therapeutic enhancement was noted following the 96 h experiment compared to either agent alone [46].

The potential for synergy using a combination of VAN has also been evaluation with non-BL antibiotics. For example, VAN in combination with clindamycin, ciprofloxacin, gentamicin, rifampicin or TMP/SMX, has been tested against hVISA and VISA strains by Kang and colleagues [47]. Combinations of VAN with ciprofloxacin, gentamicin and TMP/SMX were synergistic against some isolates of hVISA and VISA; however, there was a lack of synergy in VAN combined with either rifampicin or clindamycin, which is consistent with previous research [47,48,49]. In an evaluation conducted by Zheng and colleagues, combinations of VAN with non-BL (fosfomycin, gentamicin, rifampin and TMP/SMX) did not decrease or prevent increases of VAN MIC values during a 28-day exposure with hVISA strains. In addition, it was noted that VAN combinations with non-BL significantly increased cell wall thickness in the hVISA strain, Mu3, in comparison to VAN monotherapy [42].

Werth and colleagues performed a one-compartment PK/PD model comparing DAP + CPT to either agent as monotherapy against isolate D712 (VISA; also DNS) and therapeutic enhancement was noted following the 96 h experiment compared to either agent alone [46]. Barber and colleagues performed an evaluation of DAP plus ceftobiprole that included two VISA isolates. Following 24 h time-kill experiments, synergistic activity was noted with the combination in both strains compared to either agent alone [50]. Addition of cefazolin, cefotaxime, cefmetazole and cefepime in combination with DAP caused synergy in 100%, 100%, 87%, 80%, respectively, of tested hVISA and VISA isolates using the checkerboard method. Synergistic and bactericidal effects were further noted with DAP-cephalosporin combinations against numerous hVISA/VISA strains with conducted time-kill experiments [51].

Credito and colleagues performed an evaluation of DAP plus gentamicin or rifampin against VISA isolates (also DNS). Combination of DAP with gentamicin against VISA isolates was synergistic and showed additive activity in 66.7% and 33.3% of VISA isolates, respectively, while combination of DAP-rifampin was synergistic and showed additive activity in 33.3% and 66.7%, respectively, of VISA strains [52]. Claeys and colleagues performed in vitro time-kill experiments from 17 clinical strains of MRSA (six isolates classified as DNS, of which 5/6 (83.3%) had DAP MIC values of 4 mg/L). Following 24h experiments, synergy was noted with DAP plus TMP/SMX combination against 100% of strains [53].

### 5.2. Vancomycin-Resistant Staphylococcus aureus and Daptomycin-Non-Susceptible Staphylococcus aureus

Despite the obvious lack in efficacy with VAN monotherapy against VRSA infections, CT with VAN and BL have shown promising results in vitro and in vivo against VRSA. Similar to the “seesaw effect” observed with the utilization of VAN with anti-staphylococcal BL against other isolates of MRSA, Fox and colleagues observed the “seesaw effect” phenomenon with the use of VAN in combination with oxacillin against VRSA strain, VRS1. The authors identified that the MIC values (using Etest) of VAN and oxacillin were >256 mg/L when initially evaluated in the VRSA strain. Nevertheless, when the MIC values were tested for oxacillin in the presence of VAN (16 mg/L), the oxacillin MIC was reduced to 0.38 mg/L. Furthermore, an overnight culture of VRS1 grown in the presence of VAN at 32 mg/L exhibited an oxacillin MIC of 1 mg/L. Twenty-four hour time-kill experiments were then conducted using approximately 50% of the maximal serum antimicrobial concentrations achievable in rabbits. At eight hours, the combination of VAN and nafcillin exhibited bacterial counts that were ~5- and ~3- log_10_ CFU/mL lower compared to VAN and nafcillin alone, respectively; although, regrowth was observed at 24 h [54]. Fox and colleagues also conducted a rabbit model of endocarditis against the VRSA strain, VRS1, with VAN in combination with nafcillin. Following three days, mean (±SD) bacterial counts in log-_10_ CFU/g showed a significant reduction in bacterial load when compared to the control and the utilization of either agent as monotherapy in multiple sites (valve vegetations, kidney and spleen). Furthermore, at day seven, the bacterial counts in all three sites were significantly reduced compared to the VAN monotherapy group [54]. Tabuchi and colleagues evaluated VAN in combination with oxacillin and ceftriaxone against VRSA strains isolated by in vitro mutagenesis and VAN selection. Initially, the authors evaluated eight VRSA strains with VAN MICs between 16–32 mg/L and oxacillin/ceftriaxone MICs > 128 mg/L, in which VAN MICs were reduced to 2–8 mg/L in the presence of 2 mg/L and 4 mg/L of oxacillin and ceftriaxone, respectively. Further MIC reductions were also seen when testing VAN in the presence of seven other BL agents. Following these experiments, the combination of VAN plus ceftriaxone exhibited a therapeutic effect in a silkworm infection model with VRSA that was not observed with VAN in combination with oxacillin [55]. Synergistic activity between VAN and oxacillin has been further reported by Périchon and colleagues against numerous VRSA isolates utilizing multiple methods [56]. These data further attest to the increased activity shown with the use of VAN and BL against strains with increased MIC values to VAN.

A number of studies have explored the novel interactions between antistaphylococcal BL and DAP in *S. aureus* strains, more specifically in those expressing the DNS phenotype [57,58]. Mirroring the acclaimed “seesaw effect” observed in vitro with isolates demonstrating reduced susceptibility to VAN with the aforementioned antistaphylococcal BL, antistaphylococcal BL MIC values have been shown to decline in the presence of DAP. In a study conducted by Yang and colleagues, the authors tested five clinical isogenic strain pairs (DAP-susceptible; DAP-S/DNS) from patients that had recently failed DAP monotherapy and one pair in which the DNS strain was generated by in vitro DAP passage, to assess the “seesaw effect” phenomenon with the DAP plus oxacillin combination. The authors reported that DAP resistance was associated with enhancement in oxacillin susceptibility (three-to-four fold reductions in oxacillin MICs and population analyses susceptibility shifts (DAP curves shifted to the right (more resistant), while oxacillin curves shifted to the left (more susceptible))). Additionally, time-kill analyses demonstrated that the DNS strains became more susceptible to early killing by oxacillin when compared to their respective DAP-S parental strain and DAP plus oxacillin showed enhanced bacterial eradication in DNS strains. Furthermore, the combination of DAP plus oxacillin proved to be the most effective regimen in reducing cell densities in vegetation, kidney and spleen compared to DAP and oxacillin monotherapy in an experimental aortic infective endocarditis rabbit model in isolates expressing the DNS phenotype. It was also reported that the decline in the BL MIC values were independent of an excision of the staphylococcal cassette chromosome mec element (SCCmec), which carries mecA (the gene that encodes PBP2a), which has been previously explained as an attributing factor to a loss of methicillin resistance [57].

Furthermore, a study conducted by Dhand and colleagues also tested DAP plus antistaphylococcal BL combinations against both DAP-S strains and a DNS strain (also VISA) from clinical cases in which bacterial eradication was achieved following implementation of these combinations in persistent or refractory infections. Contrary to the decline in the BL MIC with increased DAP MIC (“seesaw effect”) shown in the aforementioned study, the authors of this study did not observe a decline in the antistaphylococcal MIC associated with a rise in DAP MIC in the DNS isolate; however, DAP MIC reductions were noted following the addition of nafcillin in their testing media [57,58]. Despite the opposing observations in regard to the “seesaw effect,” this study did observe potent synergistic activity with the DAP plus anti-staphylococcal BL combination against the DNS strain in time-kill analyses [58]. Nevertheless, the conflicting results shown between these studies indicate that the ‘‘seesaw effect” is likely not the sole mechanism responsible for the enhanced in vitro activity reported with DAP plus antistaphyloccal BL therapy. Furthermore, this study showed that DAP binding was enhanced potentially due to a reduction in membrane charge (DAP-calcium complex has overall net positive charge) in the presence of nafcillin in the DNS isolate [58]. Another study performed by Werth and colleagues noted therapeutic enhancement in a DNS strain with combination of DAP plus CPT in a one-compartment PK/PD model. Interestingly, these authors described increased binding of DAP in a DNS strain when pretreated with CPT. The authors described that CPT reduced cell wall thickness, enhanced DAP-induced depolarization and enhanced killing by human cathelicidin LL37 [46].

In a further exploration of the enhanced activity with DAP plus BL therapy, Rose and colleagues conducted in vitro experiments on a strain that eventually became DNS collected from a patient in which the DAP plus CPT combination was utilized with improved infectious outcomes. In the one-compartment PK/PD in vitro model, the authors observed enhanced activity and prevention of DAP resistance with the CT [59]. Noting that DAP nonsusceptibility has been linked to alterations in the membrane fluidity, it has been hypothesized that cationic peptides, such as DAP, exert maximal activity in the presence of a stable cell membrane [59,60]. Noting this, the authors evaluated the membrane fluidity of the isolates. Both in vitro- and in vivo-derived DAP nonsusceptibility were shown to have more fluid cell-membranes; however, the addition of CPT restored in vitro membrane fluidity to that of the initial isolate [59].

Furthermore, the utilization of DAP in combination with non-BL agents, such as rifampin, gentamicin, linezolid, TMP/SMX and tedizolid has also been explored [61,62,63,64]. Rose and colleagues discuss the concentration-dependent killing of therapeutic DAP regimens (6 mg/kg versus 10 mg/kg) against DNS strains in a simulated endocardial vegetation (SEV) PK/PD model. The authors reported the development of further resistance with the DAP 6 mg/kg monotherapy regimen against strains; although, DAP 10 mg/kg monotherapy prevented the emergence of further resistance. The addition of rifampin and gentamicin to each dosing regimen displayed enhanced in vitro activity in some strains and the suppression of further resistance was observed in the strains [61]. Steed and colleagues evaluated the utilization of DAP in combination with linezolid or TMP/SMX against two DNS strains in an SEV PK/PD model and the DAP plus TMP/SMX combination presented with the most effective activity against each strain [62]. Important to note, antagonism has been demonstrated in vitro with DAP plus linezolid and tedizolid against MRSA (although this was in DAP-susceptible strains) [63,64].

## 6. Clinical Outcome Studies

### 6.1. Beta-Lactams

#### 6.1.1. Vancomycin-Based Regimens

Dilworth and colleagues conducted one of the first real-world, clinical outcome cohort studies to evaluate the impact of BL addition to VAN in patients with MRSA bacteremia who received CT or VAN alone. The primary outcome for effectiveness was microbiological eradication of MRSA, defined as a negative blood culture obtained after therapy initiation. Eighty patients were included in the analysis with 50 and 30 patients in the CT and VAN groups, respectively. The primary outcome was achieved in 96% in the CT group compared to 80% in the VAN group (*p* = 0.021). Furthermore, CT remained favorable after adjusting for several factors in the multivariable regression model (adjusted odds ratio (aOR): 11.24; 95% confidence interval [CI], 1.7–144.3; *p* = 0.01) [65]. Despite these encouraging results, it is important to note that blood culture clearance is not necessarily a firm outcome associated with patient improvement in MRSA bacteremia and other objective clinically relevant outcomes or composite outcomes including other variables (e.g., mortality, infection recurrence) are typically more favorable. Casapao and colleagues conducted a multicenter, retrospective cohort study evaluating adult patients with MRSA bloodstream infections treated with VAN alone versus early treatment with an intravenous BL (initiated within 24 h of VAN and continued for ≥ 48 h). The primary outcome was clinical failure, which was defined as a composite of 30-day mortality, persistent bacteremia (≥7 days), relapse of bacteremia and/or alteration in antibiotic therapy due to clinical worsening. Overall, 97 patients were included in the analysis (VAN = 40 patients vs. CT = 57 patients). While not statistically significant, clinical failure was numerically lower in the CT group (30.0% and 24.6% in the VAN and CT groups, respectively; *p* = 0.552). The median duration of bacteremia also favored CT compared to VAN (4.0 (IQR, 2.5–6.5) vs. 3.0 (IQR, 2.0–5.0) days; *p* = 0.048). Furthermore, CT was inversely associated with clinical failure following multivariable analysis (aOR, 0.237; 95% CI, 0.057–0.982; *p* = 0.047) [66]. Truong and colleagues conducted a retrospective study to compare the rates of treatment failures between VAN and CT, in which included patients must have received VAN alone or in combination with a BL (> 48 h), initiated within 48 h of bacteremia onset. A total of 110 patients were included (VAN: 47 patients vs. CT: 63 patients). Combination therapy led to significantly fewer odds of treatment failure (defined as MRSA-related mortality, initiation of new anti-MRSA agent, 30-day MRSA-related readmission, lack of blood culture clearance, first negative culture from blood drawn after switching to alternative anti-MRSA agent, microbiologic relapse, and/or persistent bacteremia) when compared to VAN alone (aOR, 0.337; 95% CI, 0.142–0.997; *p* = 0.049), despite having higher APACHE-II scores and prevalence of septic shock. Importantly, 30-day all-cause mortality rates were not significantly different between the CT and VAN groups (15.0% vs. 14.9%; *p* ≥ 0.99) [67]. The impact of empiric cefepime on MRSA bacteremic patients in combination with VAN has been evaluated by Zasowski and colleagues [68]. This study evaluated 358 adults with MRSA bloodstream infection (BSI) treated with VAN alone (129 patients) versus in combination with cefepime for at least 24 h initiated within 72 h of VAN initiation (229 patients). The primary outcome was microbiologic failure, defined as bacteremia lasting seven days or more and/or recurrence of MRSA BSI within 60 days. The combination of VAN with cefepime was associated with reduced odds of microbiological failure (aOR, 0.488; 95% CI, 0.271–0.741]). Notably, VAN-cefepime combination was not associated with reduced odds of 30-day mortality (aOR, 0.952; 95% CI, 0.435–2.425) [68].

#### 6.1.2. Daptomycin-Based Regimens

The Infectious Diseases Society of America (IDSA) MRSA guidelines currently suggest using high-dose daptomycin (10 mg/kg/day), if the isolate is susceptible, in combination with another agent in the management of persistent MRSA bacteremia and/or adult patients who failed VAN therapy. Although listed agents include gentamicin, rifampin, linezolid, TMP-SMX or BL agents, BL have been the most studied [37]. One case series conducted by Dhand and colleagues evaluated MRSA BSI patients with previous VAN failure who were treated successfully with DAP 8–10 mg/kg in addition to oxacillin or nafcillin (*n* = 7). The authors stated that initial isolates were susceptible to VAN and DAP as reported by clinical microbiology reports; however, further testing of subsequent isolates in three cases reported MICs conducted via Etest ranging from (VAN: 1–4 mg/L and DAP: 0.5–4 mg/L). Although the combination resulted in rapid bacteremia clearance, this study is limited by the obvious small sample size and lack of a comparator group [58]. Moise and colleagues conducted a multicenter, retrospective observational cohort in patients with MRSA/MSSA BSI and mild-to-moderate renal impairment in 80 patients that were evaluable for effectiveness (DAP: 50 patients vs. CT: 30 patients). Cure (clinical resolution of signs/symptoms and/or no need for additional antibiotic therapy or negative culture following the end of therapy) and/or improvement (partial clinical resolution of signs/symptoms and/or need for additional antibiotic therapy to streamline/de-escalate treatment), was numerically higher in patients treated with combination DAP-BL compared to DAP monotherapy (87% vs. 78%; *p* = 0.336). The trend was more profound in BSI caused by endocarditis, bone/joint or an unknown source (90% vs. 57%; *p* = 0.061). Importantly, only two patients were reported to have a DNS isolate (hVISA/VISA phenotypes not reported). This study is limited by the small sample size and large proportion of excluded and, therefore, non-evaluable patients [69]. Jorgensen and colleagues conducted a retrospective, comparative cohort study at two academic medical centers that evaluated DAP combination with various BL agents (72 patients; primarily cefepime (43.1%) and cefazolin (25.0%)) versus DAP monotherapy (157 patients) in adults with MRSA BSI treated with DAP for ≥ 72 h and initiated ≤ 120 h of blood culture collection. Beta-lactams must have been administered for ≥ 24 h and initiated ≤ 24 h of DAP. The majority of patients (67.7%) were infected with isolates with VAN MICs ≥ 2 mg/L (although this was likely a function of having MICs potentially over-called by automated susceptibility methods), while only 2.2% of patients had isolates classified as DNS. In this study, DAP-BL was associated with significantly reduced odds of clinical failure, defined as 60-day all-cause mortality and/or 60-day recurrence (aOR, 0.386; 95% CI, 0.175–0.853). Given that DAP is an antimicrobial commonly reserved for complex infections after diagnostic susceptibility results and/or VAN failures in MRSA BSI, DAP was not the initial anti-MRSA agent in the majority of this cohort (80.3%) [70].

CPT is unique among other anti-staphylococcal BL, as it holds intrinsic in vitro activity against resistant *S. aureus* phenotypes (MRSA, hVISA, VISA, VRSA and DNS strains) [71]. McCreary and colleagues conducted a multi-center, retrospective, matched (by infection source, age and renal function) cohort study that compared patients receiving DAP-CPT ≥ 72 h (at any point in therapy) to standard-of-care, which was mostly commonly VAN (96%). A total of 58 patients in the DAP-CPT group were matched to 113 patients in the standard-of-care arm. Although not statistically significant, less patients experienced 30-day mortality in the DAP-CPT group compared to the standard-of-care group (6.8% vs. 14.2%). It was shown that a mortality benefit was numerically improved in patients with a Charlson comorbidity index ≥ 3, endovascular source and early receipts of the combination (i.e., within 72 h of index culture). Importantly, this study did not evaluate safety outcomes, which could have been integral to the ongoing debate of CT with BL and acute kidney injury (AKI) [72]. Another study conducted by Geriak and colleagues was a prospective, pilot trial that randomized patients to receive up-front DAP-CPT (17 patients) or standard-of-care with either VAN or DAP (23 patients; 91.3% VAN) as initial treatment. The initial primary outcome of the study was bacteremia duration; however, an unanticipated in-hospital mortality difference was demonstrated between the two groups favoring CT (0.0% vs. 26.1%; *p* = 0.029) and, because of the profound mortality benefit, the study was terminated early. Interestingly, bacteremia duration was not significantly different between the two groups; however, the sample size may have been too small to detect a difference. Importantly, no isolates exhibited VAN MICs ≥ 2 mg/L and all patients of which CT was used were infected with isolates with DAP MICs ≤ 0.5 mg/L [73]. Collectively and as preliminary evidence combined from McCreary and colleagues [72], DAP-CPT in fact may have an impact on mortality, if utilized in the “right” patient; however, the baseline and clinical patient characteristics that this combination may be optimized in has yet to be defined. 

#### 6.1.3. Vancomycin- or Daptomycin-Based Regimens

Few studies have evaluated CT with either VAN or DAP in addition to BL. One retrospective cohort analysis that evaluated VAN or DAP with or without a BL was conducted by Alosaimy and colleagues. This was a retrospective cohort of anti-MRSA agents (VAN or DAP) utilized as monotherapy compared to combination with any BL (98.4% being cefepime, cefazolin, CPT, piperacillin/tazobactam, ceftriaxone, ampicillin/sulbactam and meropenem). To be eligible for inclusion, BL therapy must have been initiated within 72 h of DAP or VAN initiation and continued for ≥ 24 h. The most common BL of choice was cefepime (45.9%), followed by cefazolin (33.6%). The primary outcome was a composite endpoint of clinical failure defined as: (1) 30-day mortality, (2) 60-day recurrence or (3) persistent bacteremia ≥ 5 days. Overall, 597 patients were included in the analysis (VAN/DAP monotherapy: 153 patients vs. CT: 444 patients). The results of this study showed that CT was independently associated with reduced odds of clinical failure (aOR, 0.545; 95% CI, 0.364–0.817). Importantly, the composite endpoint of clinical failure was driven by 60-day recurrence and persistent bacteremia but not 30-day mortality [74]. Another smaller cohort also evaluated patients who were treated with VAN and CPT (five cases) or DAP and CPT (six cases) for complicated MRSA bacteremia following monotherapy failure. The microbiological cure rate was 100%, with no patients experiencing bacterial relapse at 60 days. Thirty-day and 60-day all-cause mortality were found to be 11.1% and 33.3%, respectively [75].

Perhaps the most well-known trial investigating an anti-MRSA agent in addition to BL is the Combination Antibiotic Therapy for Methicillin-Resistant *Staphylococcus aureus* infection (CAMERA-II) trial, following the results of CAMERA-I trial, an open-label, multicenter, clinical trial which showed a shorter duration of bacteremia in VAN plus flucloxacillin (*n* =31) vs. VAN monotherapy (*n* = 29) [76,77]. The CAMERA-II trial was an open-label, multicenter, randomized clinical trial conducted at 27 hospital sites. The study included 352 randomized hospitalized adults with MRSA bacteremia and analyzed the primary 90-day composite outcome of mortality, persistent bacteremia at day five, microbiological relapse and microbiological treatment failure in patients with monotherapy (VAN or DAP; 178 patients) compared to CT (174 patients), with the BL of choice being intravenous flucloxacillin, cloxacillin or cefazolin. In the total study population, only ~5% of isolates exhibited VAN MICs of 2 mg/L. Patients in CT experienced a numerically lower incidence of the primary end point compared to the monotherapy arm (34.7% vs. 38.9%; absolute difference, −4.2%; 95% CI, −14.3% to 6.0%). Interesting secondary outcomes included a higher incidence of all-cause 90-day mortality in CT (20.6%) compared to monotherapy (16.1%) (difference, 4.5%; 95% CI, −3.7% to 12.7%). In addition, persistent bacteremia at day five was lower in CT (11.5%) versus monotherapy (20.4%) (difference, −8.9%; 95% CI, −16.6% to −1.2%). Notably, the study was likely underpowered (given an early termination) to detect a possible improvement in the primary endpoint in favor of CT. In addition, these results are limited to VAN plus flucloxacillin or cloxacillin, as only 13 patients received daptomycin and 27 patients received cefazolin only. It is therefore not recommended to extrapolate this clinical impact to DAP or other BLs, particularly cefazolin, or the combination of VAN-cefazolin [77].

#### 6.1.4. Nephrotoxicity with Vancomycin- or Daptomycin-Beta-Lactam Combination Therapy

One of the major concerns with CT with BL is the potential risk of nephrotoxicity. The CAMERA-II trial reported a higher incidence of AKI in the CT (23%) vs. standard therapy group (6%) (17.2%; 95% CI, 9.3–25.2%). First and foremost, VAN is an agent with well-known nephrotoxic potential and was the primary anti-MRSA agent utilized in the CT arm (98%). Secondly, only a small proportion of patients in the CT arm received cefazolin only (16%) and the safety profile of CT with cefazolin was more favorable than flucloxacillin/cloxacillin (4% vs. 27%, respectively). This raises concerns about the potential additive potential of nephrotoxicity with some but not all BL (e.g., cefazolin vs. flucloxacillin/cloxacillin/nafcillin/oxacillin vs. piperacillin/tazobactam). Lastly, VAN-AUC monitoring was not implemented in any of the VAN therapy patients in the trial. VAN-AUC monitoring is now currently recommended in the VAN dosing and monitoring guidelines, as it has been shown to reduce the incidence of nephrotoxicity when compared to trough-based monitoring [77,78].

Interestingly, other observational VAN-CT studies did not detect a higher incidence of AKI compared to the VAN monotherapy [66,74]. DAP on the other hand, is an agent that is not typically associated with nephrotoxicity as monotherapy, and, therefore, AKI was additionally not observed in most DAP-BL CT studies [69]. Interestingly, one study reported a higher incidence of AKI in the DAP-BL compared to DAP (11%) versus (3%); (*p* = 0.046). Nevertheless, the number of patients in this combination group was noticeably small with the majority (86%) receiving at least one nephrotoxic agent within 72 hours of the AKI event [70].

### 6.2. Trimethoprim/Sulfamethoxazole

#### Daptomycin-Based Regimens

There are some clinical data available to support the use of DAP in combination with TMP/SMX for invasive MRSA infections; however, data with VAN is underwhelming. One case series conducted by Claeys and colleagues was a multicenter, retrospective study in which patients (*n* =28) with MRSA infections were evaluated for effectiveness and safety of DAP plus TMP/SMX continued for ≥72 h. In this report, 92.9% of patients had a positive blood culture, with bone/joint infections being the most common primary site of infection (37.5%), followed by deep abscess (32.1%), skin/soft tissue (28.5%) and endocarditis (25.0%). The majority of isolates exhibited VAN MICs of 2 mg/L (60.7%), while 21.4% of isolates were classified with the DNS phenotype (83.3% with DAP MIC of 4 mg/L). The median time to clearance of bacteremia was 2.5 (IQR, 1.0–6.8) days following CT initiation (in those who had not cleared prior to or at the time of initiation of CT), with the majority of patients achieving microbiological eradication (85.7%). Twenty one percent of patients experienced an adverse drug reaction (three cases each of creatine phosphokinase elevation and hyperkalemia) [53]. Another case series that reported the effectiveness of CT with DAP plus TMP/SMX was published by Avery and colleagues in two patients with a history of a MRSA infections. Both patients presented with DNS/VISA bacteremia secondary to vertebral osteomyelitis and were treated successfully with the combination [79]. A case report also successfully treated a patient with MRSA BSI secondary to mitral valve endocarditis with a large cardiac vegetation with DAP plus TMP/SMX CT. The combination led to clinical improvement and cardiac mitral vegetation clearance [80].

### 6.3. Rifampin

#### 6.3.1. Vancomycin-Based Regimens

The IDSA MRSA guidelines recommend that use of rifampin should be in combination with other antibiotics, rather than monotherapy (due to rapid development of resistance when used as monotherapy) [37]. The current limited evidence available exhibits that the use of rifampin increases drug interactions and the possibility of adverse effects [81]. A cohort analysis from a randomized trial was conducted by Levine and colleagues in which 42 MRSA endocarditis patients received either VAN alone (*n* = 22) or in combination with rifampin (*n* = 20) for 28 days. The median duration of bacteremia was numerically higher in the CT arm compared to monotherapy arm (nine vs. seven days), the median duration of fever was seven days in both groups and clinical cure was numerically higher in CT vs. monotherapy groups (90% vs. 82%, *p* ≥ 0.20) [82]. A randomized, prospective, open-label study was conducted by Jung and colleagues in which 83 patients in the medical intensive care unit with nosocomial MRSA pneumonia were randomized to receive either VAN alone (*n* = 42) or in combination with rifampin (*n* = 41). Clinical cure rates were higher in the CT versus VAN monotherapy group in the modified intention-to-treat population (53.7% and 31.0%; *p* = 0.047), while 60-day mortality was higher in the VAN monotherapy arm (50.0% vs. 26.8%; *p* = 0.042). However, total adverse events were numerically higher in the CT arm, with hyperbilirubinemia being the most common (13%). The authors noted that no isolates exhibited VAN MICs > 2 mg/L [83]. One retrospective, matched (by time of diagnosis) cohort evaluated patients with MRSA definite native valve endocarditis in patients treated with VAN versus VAN plus rifampin and found that 30-day survival was higher in VAN monotherapy vs. CT (95.2% and 78.6%; *p* = 0.048) and median duration of bacteremia being higher in the CT group (5.2 (range, 1.0–26.0) vs. 2.1 (range, 1.0–8.0) days; *p* < 0.001). Importantly, valve surgery occurred more commonly in the CT arm (21.4% vs. 4.8%; *p* = 0.03). Rifampin-resistant isolates emerged in 21.4% of those receiving CT, while hepatotoxicity was more common in the CT group (21.4% vs. 2.4%; *p* = 0.014). It is important to note that VAN MICs were not reported [84].

#### 6.3.2. Daptomycin-Based Regimens

Daptomycin has been used with success in MRSA bacteremia caused by a DNS isolate with the addition of rifampin. In this case, an 84 year-old male presented with infectious symptoms following cystoscopy. Following ten days of persistent bacteremia while on VAN (initial VAN and DAP MICs: ≤ 1 mg/L and 0.25 mg/L, respectively), therapy was changed to DAP; however, despite DAP monotherapy for an additional three days, the patient remained bacteremic. Six days later, the DAP MIC increased to 2 mg/L and rifampin was initiated the following day. Two days following rifampin initiation, blood cultures were negative and, following discharge and a six-week course of DAP + rifampin, the patient was doing well four months following therapy [85].

While DAP is not recommended for the management of pneumonia due to sequestration by pulmonary surfactant within the bronchoalveoli, clinical evidence suggests that it may be effective as monotherapy for the management of septic pulmonary emboli (SPE) [86,87]. However, a case series of four patients with MRSA bacteremia and SPE in the absence of endocarditis, DAP and rifampin was used with clinical success in patients who failed therapy with VAN. All isolates reported in this case series had VAN MICs of 2 mg/L and DAP MICs ≤ 1 mg/L [88].

### 6.4. Linezolid

#### 6.4.1. Vancomycin-Based Regimens

Use and/or addition of a toxin inhibiting antibiotic, such as linezolid, may be beneficial in suppressing staphylococcal toxin production [89]. However, to our knowledge, no combination studies have been conducted to evaluate the impact of VAN plus linezolid in the clinical realm. Notably, an in vitro and in vivo studies have demonstrated that these two antibiotics demonstrate antagonism [64,90].

#### 6.4.2. Daptomycin-Based Regimens

Daptomycin has been used in combination with linezolid and rifampin in a patient with a MRSA medical device infection which was complicated by bacteremia, meningitis and osteomyelitis. The isolate exhibited VAN and DAP MICs of 1 mg/L and 0.5 mg/L, respectively and the patient’s blood cultures showed clearance within four days after the addition of linezolid and two days after the addition of rifampin to DAP [91]. Another case reported the successful use of DAP plus linezolid for the management of MRSA native tricuspid valve endocarditis following unsuccessful use of VAN and DAP plus gentamicin with the isolates exhibiting a VAN MIC of 4 mg/L and DAP MIC of 4 mg/L. The cultures of this patient cleared after 13 days of CT. Importantly, the regimen was switched to linezolid and gentamicin due to DAP resistance, although this was after the patient cleared [92]. Similarly, another case reported success with DAP plus linezolid used as salvage therapy for the treatment of MRSA right-sided infective endocarditis with concomitant septic pulmonary embolism (VAN and DAP MICs both reported to be 1 mg/L) [93]. Another case of salvage therapy with DAP plus linezolid (and meropenem) in a patient case with persistent MRSA bacteremia has also been reported. Although the patient did not clear their blood cultures after three and seven days of DAP monotherapy and DAP-CPT, respectively, the patient ultimately cleared one day after the previously listed salvage therapy was initiated [94]. It is important to note that in vitro studies have demonstrated antagonism with the combination of DAP and linezolid, so synergy and antagonism is likely strain dependent [64].

## 7. Conclusions

Invasive MRSA infections lead to significant morbidity and mortality. Despite VAN being used as one of the gold standard treatments for decades, no therapy has been proven to be superior. Daptomycin is a common agent utilized up-front or in VAN failures, however the prevalence of resistance in *S. aureus* to both VAN and DAP is increasing. Combination therapy, particularly with BL, has been advocated in certain clinical scenarios following numerous in vitro and in vivo data showing enhancements in bacterial killing and/or the prevention of VAN/DAP resistance or the reduction of further resistance in VAN/DAP strains with resistant phenotypes. There have been numerous clinical studies evaluating CT of VAN/DAP with various antibiotics and several primary infections, with inconsistent results. Despite the studies to date, BL antibiotics seem to be the most promising to be utilized in combination with the gold-standards for invasive MRSA infections (results summarized in Table 1). Despite the potential enhancement in outcomes, it is very important to further evaluate the safety of CT. Further randomized clinical trials are needed to evaluate if CT regimens with VAN or DAP may prove to be superior to VAN and/or DAP monotherapy in optimizing patient outcomes and preventing the emergence of resistance.

## Figures and Tables

**Table 1:** Clinical Outcomes of Glycopeptides in Combination with Beta-Lactams from Selected Studies.

Study	Anti-MRSA Combination Agent	CT Agent	Overall Population and Outcome	Results
Dhand (2011) [58]	DAP	Nafcillin or oxacillin	Patients with persistent MRSA bacteremia (7–22 days) refractory to VAN changed to DAP and nafcillin or oxacillin (*n* = 7) evaluated for blood sterilization	Blood sterilization within 24–48 h achieved in 100% of patients
Moise (2013) [69]	DAP	BL (not specified)	Patients with mild-to-moderate renal dysfunction with S. aureus bacteremia (MRSA/MSSA) receiving DAP monotherapy (*n* = 50) or DAP plus BL (*n* = 30) were evaluated for cure (clinical resolution of signs/symptoms and/or no need for additional antibiotic therapy or negative culture following the end of therapy) and/or improvement (partial clinical resolution of signs/symptoms and/or need for additional antibiotic therapy to streamline/de-escalate treatment)	Outcome benefit rates were numerically higher in the CT group vs. DAP monotherapy (87% vs. 78%, *p* = 0.336)
Dilworth (2014) [65]	VAN	Piperacillin/ tazobactam, cephalexin, cefazolin, cefoxitin, ceftriaxone, ceftazidime, cefotaxime, cefepime, imipenem, meropenem, ampicillin, nafcillin, amoxicillin/clavulanate	Patients with MRSA BSI receiving VAN monotherapy (*n* = 30) or VAN plus beta-lactam (*n* = 50) were evaluated for microbiological eradication (negative blood culture obtained after initiation of therapy)	Microbiological eradication was higher in CT versus VAN monotherapy (96% vs. 80%, *p* = 0.021)
Casapao (2017) [66]	VAN	Ampicillin, nafcillin, oxacillin, ampicillin/sulbactam, piperacillin/tazobactam, cefazolin, cefoxitin, ceftriaxone, ceftazidime, cefotaxime, cefepime, imipenem/cilastatin, doripenem, ertapenem, meropenem	Patients with MRSA BSI receiving VAN monotherapy (*n* = 40) or VAN plus early adjuvant BL (*n* = 57) were evaluated for clinical failure (composite of 30-day mortality, persistent bacteremia (≥ 7 days), bacteremia relapse or change in antibiotic therapy due to clinical worsening)	Clinical failure was inversely associated with receipt of CT (aOR, 0.237, 95% CI (0.057–0.982))
Truong (2018) [67]	VAN	BL (unspecified), except ceftazidime and aztreonam	Patients with MRSA BSI receiving VAN monotherapy (*n* = 47) or VAN plus BL (*n* = 63) were evaluated for treatment failure (composite of clinical [initiation of new anti-MRSA agent(s), MRSA-related mortality, and/or 30-day MRSA-related readmission] and microbiologic failure [lack of bacteremia clearance, first negative blood culture drawn after switching to alternative anti-MRSA regimen, microbiologic relapse, and/or persistent (> 5 days) bacteremia])	Treatment failure was inversely associated with receipt of CT (aOR, 0.337, 95% CI (0.142–0.997))
Zasowski (2019) [68]	VAN	Cefepime	Patients with MRSA BSI receiving VAN monotherapy (*n* = 129) or VAN plus cefepime (*n* = 229) were evaluated for microbiological failure (bacteremia ≥ 7 days and/or 60-day recurrence).	VAN plus cefepime was associated with reduced odds of microbiological failure (aOR, 0.488, 95% CI, (0.271–0.741)) but was not associated with reduced odds of 30-day mortality (aOR, 0.952, 95% CI (0.435–2.425)).
Jorgensen (2019) [70]	DAP	Cefepime, cefazolin, ceftaroline, ceftriaxone, meropenem, piperacillin-tazobactam, ertapenem, ampicillin-sulbactam	Patients with MRSA BSI receiving DAP monotherapy (*n* = 157) or DAP plus BL (*n* = 72) were evaluated for clinical failure (composite of 60-day all-cause mortality and/or 60-day recurrence)	Clinical failure was inversely associated with receipt of CT (aOR, 0.386, 95% CI, 0.175–0.853)
Geriak (2019) [73]	DAP	Ceftaroline	Patients with MRSA BSI receiving VAN or DAP (*n* = 23) or DAP plus CPT (*n* = 17) were evaluated for bacteremia duration and in-hospital mortality	DAP plus CPT was not associated with significantly lower bacteremia duration (CT: 3.0 [1.5–5.5] vs. MT: 3.0 (1.0–5.3) days; *p* = 0.56) but was associated with lower in-hospital mortality (CT: 0% vs. MT: 26%; *p* = 0.03)
McCreary (2019) [72]	DAP	Ceftaroline	Patients with MRSA BSI receiving VAN or DAP (*n* = 113) or DAP plus CPT (*n* = 58) were evaluated for bacteremia duration and 30-day mortality	MT was associated with lower bacteremia duration (CT: 9.3 vs. MT: 4.8 days; *p* < 0.001) and CT was not associated with significantly lower 30-day mortality (CT: 6.8% vs. MT: 14.2%; *p* > 0.05)
Tong (2020) [77]	VAN or DAP	Flucloxacillin, cloxacillin, cefazolin	Patients with MRSA BSI randomized to receive VAN/DAP (*n* = 178) or CT (*n* = 174) were evaluated at 90-days for a composite of mortality, persistent bacteremia at day 5, microbiological relapse (MRSA positive blood culture ≥ 72 h after a previous negative culture) and microbiological treatment failure (positive MRSA sterile site culture ≥ 14 days following randomization)	Primary composite end point was numerically less frequent in the CT compared to MT group (35% vs. 39%, absolute difference, -4.2%; 95% CI, −14.3% to 6.0%)
Alosaimy (2020) [74]	VAN or DAP	Cefepime, cefazolin, ceftaroline, piperacillin/tazobactam, ceftriaxone, ampicillin/sulbactam, meropenem, aztreonam, other unspecified carbapenems and cephalosporins	Patients with MRSA BSI receiving VAN or DAP (*n* = 153) or VAN/DAP plus BL (*n* = 444) were evaluated clinical failure (composite of 30-day mortality, 60-day recurrence or persistent bacteremia (> 5 days))	Clinical failure was inversely associated with receipt of CT (aOR, 0.545, 95% CI, 0.364–0.817)

Abbreviations: BL: beta-lactam; BSI: bloodstream infections; CT: combination therapy; CPT: ceftaroline; DAP; daptomycin; MT: monotherapy; MRSA: methicillin-resistant *Staphylococcus aureus*; MSSA: methicillin-susceptible *Staphylococcus aureus*; VAN: vancomycin.
